# Multisensory Technology for Flavor Augmentation: A Mini Review

**DOI:** 10.3389/fpsyg.2018.00026

**Published:** 2018-01-30

**Authors:** Carlos Velasco, Marianna Obrist, Olivia Petit, Charles Spence

**Affiliations:** ^1^Center for Multisensory Marketing, Department of Marketing, BI Norwegian Business School, Oslo, Norway; ^2^Sussex Computer Human Interaction Lab, Creative Technology Research Group, School of Engineering and Informatics, University of Sussex, Brighton, United Kingdom; ^3^INSEEC Business School, Paris, France; ^4^Crossmodal Research Laboratory, Department of Experimental Psychology, University of Oxford, Oxford, United Kingdom

**Keywords:** food, multisensory, experience, augmentation, vision, audition, touch, haptics

## Abstract

There is growing interest in the development of new technologies that capitalize on our emerging understanding of the multisensory influences on flavor perception in order to enhance human–food interaction design. This review focuses on the role of (extrinsic) visual, auditory, and haptic/tactile elements in modulating flavor perception and more generally, our food and drink experiences. We review some of the most exciting examples of recent multisensory technologies for augmenting such experiences. Here, we discuss applications for these technologies, for example, in the field of food experience design, in the support of healthy eating, and in the rapidly growing world of sensory marketing. However, as the review makes clear, while there are many opportunities for novel human–food interaction design, there are also a number of challenges that will need to be tackled before new technologies can be meaningfully integrated into our everyday food and drink experiences.

## Introduction

Interest in multisensory perception is growing rapidly in the fields of Human–Computer Interaction (HCI, Obrist et al., 2016), sensory marketing (e.g., Petit et al., 2015), and the arts (e.g., Haverkamp, 2013; Vi et al., 2017). This places knowledge concerning the human senses, and their interactions, at the center of design processes (Obrist et al., 2017). In the context of Human-Food Interaction (HFI, Choi et al., 2014; Comber et al., 2014), there has been an increasing interest in how multisensory technologies can augment/modify multisensory flavor perception^1^, and food and drink experiences more generally and possibly also to sensorially nudge people toward healthier food behaviors (Nijholt et al., 2016; Petit et al., 2016). The key idea here is that flavor is a multisensory construct (involving taste, or gustation, olfaction, and possibly also trigeminal components; see Kakutani et al., 2017) and all the senses can potentially influence the way in which we experience it (Spence, 2015a). Hence, multisensory technologies, that is, technologies that are designed to stimulate the human senses, allow researchers to control the different inputs that accompany a given multisensory flavor, or food experience.

Why, it can be asked, use multisensory technologies in order to augment our flavor experiences? Given that technology is already ubiquitous in our everyday experiences, such technologies in the context of HFI hold the potential to transform how we will eat in the future (Spence and Piqueras-Fiszman, 2013). More specifically, we want to argue that a meaningful marriage of multisensory science (e.g., considering the guiding principles of multisensory flavor perception, e.g., Prescott, 2015; Spence, 2015a) and technology in systems capable of augmenting flavor perception can impact what people choose to eat and drink, how they perceive the ensuing flavor experience, and how much they ultimately end-up consuming.

In this mini-review, we present an overview of multisensory technologies for flavor augmentation that have been developed recently. Importantly, we follow Prescott’s (2015) distinction between core intrinsic (taste, smell, and some elements of touch) and extrinsic (e.g., color, shape, atmospheric sound – which can modulate the experience of flavor but might not be constitutive) elements of the flavor experience and focus on the role of the latter in flavor augmentation^2^. Our aim is to make researchers working in different fields aware of the various ways in which multisensory technologies that target extrinsic elements of flavor experiences are starting to transform how we interact with and experience what we consume. As such, we expect this manuscript to provide a first point of contact for those interested in multisensory technologies and flavor augmentation. Additionally, we hope that this review will contribute to bridging the gap between researchers working in the fields of HCI/HFI on the one hand, and food science, marketing, and psychology, on the other. It is our view that the latter disciplines would benefit from an increased awareness of the different technologies that are currently available to those working in HCI/HFI. These latter, in turn, would realize some of the potential uses that their technologies have, as well as the financial gains that may derive from such applications. We conclude by presenting challenges that face those wanting to augment flavor perception and experiences.

## Flavor Perception and Augmentation

Here, we present some key concepts and technologies associated with flavor augmentation on the basis of flavor extrinsic cues (see **Table 1** for a summary of some representative examples). People rarely put something in their mouth without first having made a prediction about what it will taste like. These expectations, set primarily by what we see and smell (orthonasally), but also sometimes by what we hear and feel/touch, anchor the experience when we come to taste something (see Verhagen and Engelen, 2006). For example, visual cues such as color or shape can be used to guide food and drink expectations, search, and augmentation based on semantic knowledge (learned associations as a function of a common identity or meaning such as between the color red and tomato flavor) and crossmodal correspondences (feature compatibility across the senses, such as between sweetness and curvature; e.g., Shermer and Levitan, 2014; Velasco et al., 2015, 2016b; Sawada et al., 2017).

**Table 1:** Examples of multisensory technologies for flavor augmentation based on extrinsic cues associated with the flavor and food/drink experiences.

Augmentation	Technology	What does it allow to control?	Reference
Visual	Projective-AR	Food color and texture	Nishizawa et al., 2016
	Project Nourished (VR)	Eating environments (and overall multisensory experience)	http: //www.projectnourished.com/ (To the best of our knowledge, there are not studies published associated with this project)
Auditory	Chewing Jockey	Sounds associated with mastication	Koizumi et al., 2011
	EducaTableware	Cutlery sounds	Kadomura et al., 2013
Tactile/haptic	Gravitamine spice	Cutlery weight	Hirose et al., 2015
	Vibration system	Vibrations associated with beverage pouring	Ikeno et al., 2015
Multi-sense	Straw-like User Interface (SUI)	Pressure, vibration, and sound during drinking	Hashimoto et al., 2006
	Audio-haptic rendering	Vibrations and sounds during beverage pouring	Ikeno et al., 2013

### Visual Augmentation

Vision is critical when it comes to setting our flavor expectations and hence modifying our flavor experiences (Piqueras-Fiszman and Spence, 2015; Spence et al., 2016). Current technologies allow one to go beyond traditional means of food enhancement, based on vision (e.g., just matching or mismatching visual information with a given flavor), and to create novel HFIs that dynamically modulate our flavor experiences, and perhaps also more broadly, our consumption behaviors.

For instance, Nishizawa et al. (2016) developed an augmented reality (AR) system using a projector and a camera in order to transform the visual characteristics (e.g., texture, color) of foods or plates digitally, in real-time, based on the evidence showing that these factors influence people’s perception of what they eat (e.g., Okajima et al., 2013). In a similar vein, and as a more specific example, Okajima and Spence (2011) modified the texture of tomato ketchup by changing the skewness of the luminance histogram whilst not changing the chromaticity of the video feed. Modifying such visual features, among others, was found to influence sensory attributes such as the ketchup’s perceived consistency and taste such that different skewness led participants think they were tasting different ketchups (see also Narumi et al., 2011; Huisman et al., 2016).^3^

Augmented reality systems build on mixed reality (MR) interactions (i.e., incorporating both virtual and real inputs, see Narumi, 2016). AR would appear to have been adopted more rapidly than virtual reality (VR) in flavor- and food-related technology research and practice. For instance, Kabaq^4^ is an AR food program that offers restaurants the option of presenting their customers with 3D visions of the food that they serve, before ordering. As for VR, whilst some researchers are exploring the possibility of virtual flavors via digitally controller electric and thermal taste sensations, such systems are currently of very limited use/potential (see Spence et al., 2017, for a critique). That being said, there is potential to design experiences in VR that target the user’s flavor expectations (e.g., before going to a restaurant or buying a product). There are currently many ongoing research initiatives that have been designed to further our knowledge on the applications of VR systems to flavor/food experience design^5^ (e.g., Bruijnes et al., 2016). One such initiative involves using VR to expose (virtually) people with food-related medical conditions to obesogenic environments (Schroeder et al., 2016; Wiederhold et al., 2016).

Companies are now exploring product packaging that, together with a smartphone, can be turned into inexpensive VR headsets (e.g., as in the case of some of Coca Cola’s cardboard packaging). Such headsets might enable brands to deliver targeted experiences in VR. Whilst, at present, this approach appears more as a curiosity than anything else, we anticipate that it might 1 day become an extension of the total product experience, in that any given product might have its own customized multisensory experience(s) in VR (Lingle, 2017; Michail, 2017). Such experiences may be designed based on research showing the influence of visual atmospheric cues (e.g., lightning, environment) on flavor perception (Stroebele and De Castro, 2004; Spence et al., 2014).

### Auditory Augmentation

Often described as the forgotten flavor sense, research on auditory contributions to the experience of eating and drinking has grown rapidly in recent years. The evidence currently suggests that audition is critical to the perception of attributes such as crunchiness, crispiness, and crackliness (Spence, 2015b). What is more, the sounds associated with eating and drinking such as chewing, gulping, or lip-smacking (Zampini and Spence, 2004; Youssef et al., 2017), environmental noise (Woods et al., 2011), and soundscapes/music (Crisinel et al., 2012; Kantono et al., 2016) can all influence food perception (e.g., tastes, odors, textures, flavors). For instance, noise can enhance the perception of umami and diminish perceived sweetness (Yan and Dando, 2015). Based on these kinds of findings, there is growing interest in developing technologies that can capitalize on the sense of audition for flavor augmentation (Velasco et al., 2016a; see also a reference to “EverCrisp app” by Kayac Inc in Choi et al., 2014, designed to enhance food-biting sounds).

Systems that build on the role of mastication sounds on flavor perception constitute one example of flavor augmentation based on audition. The “Chewing Jockey,” for example, is a device that uses a bone-conduction speaker, a microphone, jaw movement tracking sensor, and a computer, to allow one to monitor mastication and use such movements to synchronize and control sound-delivery (Koizumi et al., 2011). Based on such a concept, researchers are now interested in the modulation of texture perception (e.g., in the elderly who find it difficult to chew solid foods, see Endo et al., 2016), consumption monitoring (Elder and Mohr, 2016), and the creation of novel and fun food interactions (e.g., mapping unexpected sounds such as screaming sounds to gummies chewing, Koizumi et al., 2011), by modifying, or replacing the actual sounds of mastication.

The role of audition goes beyond mastication sounds though, as there are many other auditory cues that we may hear at more or less the same time as we eat (Velasco et al., 2016a). These include those sounds directly associated with our interaction with the food, but also atmospheric sounds. In terms of the former, Kadomura et al. (2013) introduced “EducaTableware,” which include a fork and a cup that use food’s (electrical) resistance values, and eating times and intervals to emit sounds while a user consumes a given food (see also Kadomura et al., 2011). This device creates a novel interaction between the user and the food (e.g., for entertainment). In terms of atmospheric sounds, although music devices are ever-present, there is much room for development. For example, based on the idea that sounds can influence taste/flavor perception and enjoyment (i.e., hedonics; Spence, 2017), MR systems that combine real food and audiovisual virtual environments may be developed (e.g., what would it be like to eat a cheesecake, via VR, on Mars? see Project Nourished^6^).

### Tactile/Haptic Augmentation

What we feel/touch can also influence the perception of flavor while eating and drinking (e.g., Krishna and Morrin, 2008; Biggs et al., 2016; Slocombe et al., 2016). Researchers have demonstrated that elements such as the weight, size, shape of cutlery and tableware can influence flavor expectations and perception (Spence, 2017; van Rompay et al., 2017). An example of this comes from Michel et al. (2015), who reported that relatively heavy cutlery can lead to tastier food perception. Notably, similar to systems that build on vision and audition, most of the potential of touch-related devices for flavor augmentation so far has been in terms of MR solutions.

For instance, Hirose et al. (2015) developed a fork-type device that involves an accelerometer, a photo reflector sensor, and motor slider, to digitally control the center of gravity, and therefore the perceived weight, of the eating utensil. The intention behind “Gravitamine spice” is to modify the felt weight of the food/cutlery before eating. Another example comes from Ikeno et al. (2015) who showed that different patterns of vibrations accompanying the action of pouring a beverage can influence how much is poured. These technologies might potentially be used to nudge people to consume a little less, to create novel human–food interactions, and to augment flavor. Meanwhile, Iwata et al. (2004) developed a haptic device for biting, known as the “Food simulator.” This interface generates a force on the user’s teeth, which is based on the force profile of people biting a given food, in order to stimulate the sensation of biting such a food.

There are also multiple emerging haptic/tactile technologies that can be used for flavor augmentation or innovative HFI design. For instance, Tsutsui et al. (2016) developed a high resolution tactile interface for the lips, a part of the body that is often stimulated while eating and drinking, which created a new interaction design space. There might also be opportunities when it comes to MR scenarios where people eat and receive haptic feedback on their body either associated with the food they eat (e.g., Choi et al., 2014) or remote dining with touch-related signals from co-diners (e.g., Wei et al., 2011). Of course, in many cases, there may be no specific need for haptic/tactile interfaces be technology-based. Nevertheless, what technology can potentially offer is a new way of stimulating the skin (e.g., contingently) and therefore opens-up a space for novel interactions and flavor experiences.

### Combining Multiple Extrinsic Flavor Elements for Flavor Augmentation

Visual, auditory, and tactile/haptic flavor augmentation systems have, in general, focused on allowing the integration of one property (e.g., color) or series of properties (e.g., color and shape), in a given sense (e.g., vision) with specific flavor experiences. Importantly, though, eating and drinking constitute some of life’s most multisensory experiences (e.g., involving color, shape, sound, vibration, texture roughness, etc., Spence, 2015a). It is perhaps little wonder, then, that those trying to emulate more real-life experiences have focused on designing technologies that allow the integration and controllability of inputs associated with multiple sensory modalities (e.g., Kita and Rekimoto, 2013). For example, the “Straw-like User Interface (SUI)” augments the user’s drinking experiences based on multisensory inputs (e.g., using pressure, vibration, and sound, see Hashimoto et al., 2006; see also Ranasinghe et al., 2014). Another example comes from Ikeno et al. (2013) who developed a system that combines vibrations and sounds (e.g., an auditory “glug” characteristic of a Sake bottle when a drink is poured) to influence the subjective impression of a liquid. Whilst there is certainly no need to stimulate all of the senses, for a given flavor augmentation, solutions that allow the delivery of multiple cues at a given time might broaden the scope for multisensory design.

## Discussion and Conclusion

This review presents flavor, and more general, food and drink augmentation in the context of multisensory experience design. In particular, we provide an overview of both older and more recent efforts around flavor augmentation in HFI. In addition to psychologists and sensory/food scientists, those researchers involved in HCI are increasingly exploring new ways of transforming our eating and drinking experiences. The proliferation of VR and MR systems provide the most promising platforms for new (multisensory) flavor experiences in the near future.

We have concentrated on exemplar systems that have capitalized on flavor extrinsic elements from vision, audition, and touch/haptics for flavor augmentation. Whilst such systems are still far from ubiquitous, they are nevertheless increasingly being considered by some of the key players/influencers of the food and drink industry – such as, for example, chefs, culinary artists, experiential brand event managers, and so on (Spence, 2017).

Importantly, however, there are multiple challenges ahead for both researchers and practitioners who may be interested in using multisensory technologies for flavor augmentation. First, a vast gap often exists between technology, as showcased in HCI research, and what actually ends-up in more commercial settings relevant to those working in the food and drink industry (e.g., in a fancy modernist restaurant on in a branded experiential event). Second, there is a need for long-term follow-up investigations, as most of the research examples that have been reported to date have been based on one-off, small scale studies (e.g., small sample sizes with limited experimental designs; for example, Nishizawa et al., 2016, conducted two studies with four and six participants, respectively). There is a need to control for variables such as novelty and habituation, something that will undoubtedly be required in order to know whether the brain adapts to the multisensory flavor experiences designed with new technologies, or whether instead the benefits may last into the medium/longer-term. In other words, there needs to be a consistent added value for flavor and food augmentation to become more than a one-time curiosity or gimmick.

The aforesaid challenges might be addressed (at least in part), by the meaningful integration of scientific insights concerning multisensory flavor perception with new technologies. Whilst research on the principles governing multisensory integration during flavor perception is ongoing (see Prescott, 2015; Spence, 2015a), design guidelines have nevertheless been suggested (Schifferstein and Desmet, 2008; Velasco et al., 2016a). Taking a full-scale, evidence-based approach to the design of multisensory flavor experiences that incorporates technology is not an easy task and therefore will require both time and a fundamentally multidisciplinary approach.

However, the hope is that multisensory technologies might inspire tomorrow’s practitioners to: (1) modify flavor perception and experiences; (2) nudge people toward healthier food behaviors; (3) facilitate food choice before ordering/buying; (4) make dining more entertaining. For example, TeamLab, an art collective, collaborated recently with the Sagaya restaurant in Tokyo to develop a dining experience described as follows: “*when a dish is placed on the table, the scenic world contained within the dish is unleashed, unfolding onto the table and into the surrounding space. For example, a bird painted on a ceramic dish is released from the dish and can perch on the branch of a tree that has been unleashed from a different dish*” (cited in Stewart, 2017, p. 1^7^). Other examples include the oft-mentioned Michelin-starred modernist restaurant Ultraviolet by Paul Pairet in Shanghai. There, diners are guided through a multisensory dining experience that is accompanied by changing lights, projections, and soundscapes (Yap, 2016; Spence, 2017). Technology in the context of multisensory flavor experience design is a means to transform sensory information into ingredients/raw materials for our future flavor experiences. In that sense, we foresee more applications and novel design spaces being explored in the wider food and drink world.

## Author Contributions

All authors listed have made a substantial, direct and intellectual contribution to the work, and approved it for publication.

## Conflict of Interest Statement

The authors declare that the research was conducted in the absence of any commercial or financial relationships that could be construed as a potential conflict of interest.

## Footnotes

1 By flavor augmentation, we refer to the process of modifying, boosting, or enhancing, a given flavor experience, be it perceived or imagined, using technology.

2 Whilst we do not focus on olfactory interfaces, devices based on orthonasal olfaction (and its interaction with other senses) have also been proposed by researchers working in the topic of flavor augmentation (e.g., Nambu et al., 2010; Hashimoto and Nakamoto, 2016).

3 See also Okajima’s Laboratory website (https://goo.gl/kH1S9Q) for some examples.

4 http://www.kabaq.io/

5 The ACM International Conference on Multimodal Interaction workshop on “Multisensory Human-Food Interaction” (https://goo.gl/HRRdVs) or the Special Issue on “Virtual reality and food: Applications in sensory and consumer science” in The Journal of Computers and Graphics (https://goo.gl/FKwWjF).

6 http://projectnourished.com/

7 See also a report in DesignBoom with visual documentation: https://www.designboom.com/design/teamlab-interactive-saga-beef-restaurant-sagaya-ginza-tokyo-04-13-2017/.
